# Epidemiologic Methods of Assessing Asthma and Wheezing Episodes in Longitudinal Studies: Measures of Change and Stability

**DOI:** 10.2188/jea.JE20120201

**Published:** 2013-11-05

**Authors:** Nelís Soto-Ramírez, Ali H. Ziyab, Wilfried Karmaus, Hongmei Zhang, Ramesh J. Kurukulaaratchy, Susan Ewart, Syed Hasan Arshad

**Affiliations:** 1 Division of Epidemiology, Biostatistics, and Environmental Health, University of Memphis, Memphis, Tennessee, United States; 2 Department of Epidemiology and Biostatistics, University of South Carolina, Columbia, South Carolina, United States; 3 David Hide Asthma and Allergy Research Centre, Isle of Wight, and University of Southampton, Southampton, United Kingdom; 4 Michigan State University, East Lansing, Michigan, United States

**Keywords:** asthma, cohort study, incidence, prevalence, trajectory, transition probability

## Abstract

**Background:**

In settings in which diseases wax and wane, there is a need to measure disease dynamics in longitudinal studies. Traditional measures of disease occurrence (eg, cumulative incidence) do not address change or stability or are limited to stable cohorts (eg, incidence) and may thus lead to erroneous conclusions. To illustrate how different measures can be used to detect disease dynamics, we investigated sex differences in the occurrence of asthma and wheezing, using a population-based study cohort that covered the first 18 years of life.

**Methods:**

In the Isle of Wight birth cohort (*n* = 1456), prevalence, incidence, cumulative incidence, positive and negative transitions, and remission were determined at ages 1 or 2, 4, 10, and 18 years. Latent transition analysis was used to simultaneously identify classes of asthma and wheezing (related phenotypes) and characterize transition probabilities over time. Trajectory analysis was used to characterize the natural history of asthma and wheezing.

**Results:**

Regarding time-specific changes, positive and negative transition probabilities were more informative than other measures of associations because they revealed a sex switchover in asthma prevalence (*P* < 0.05). Transition probabilities were able to identify the origin of a sex-specific dynamic; in particular, prior wheezing transitioned to asthma at age 18 years among girls but not among boys. In comparison with latent transition analysis, trajectory analysis did not directly identify a switchover in prevalence among boys and girls.

**Conclusions:**

In longitudinal analyses, transition analyses that impose minimal restrictions on data are needed in order to produce appropriate information on disease dynamics.

## INTRODUCTION

Asthma is a chronic disorder that accounts for considerable morbidity in children and adults. Major hallmarks contributing to the heterogeneity of clinical manifestations of asthma include airway inflammation, airway obstruction, and bronchial hyper-responsiveness.^1^ Recently, wheezing and asthma have been recognized as distinct phenotypes, with possibly different clinical prognoses.^1^^,^^2^ Gender has a central role in the natural history of asthma during childhood and adolescence: before puberty the disorder predominantly affects boys, whereas girls are more likely to be affected during adolescence and early adulthood.^3^^–^^5^

Population-based longitudinal studies provide an unmatched opportunity to investigate the natural history of diseases. However, the ability of different epidemiologic measures of disease occurrence to detect differences among groups (eg, sex differences) is highly dependent on the method used. The lack of appropriate measures to describe the remitting–relapsing course and pattern of diseases hampers epidemiologic research. Traditional measures of occurrence (eg, prevalence, incidence, and cumulative incidence) do not address changes and stability or are limited to stable cohorts, which may lead to erroneous conclusions due to exclusion of individuals with incomplete follow-up information. To elucidate the effect of sex on the time-course of asthma and wheezing and evaluate the performance of epidemiologic measures, we used data from the Isle of Wight birth cohort, which covers the first 18 years of life. In this report we compare the results of different measures of disease occurrence and the ability of such measures to detect age-specific changes in asthma and wheezing occurrence. Latent transition analysis was used to determine the dynamics of defined phenotypes over time, and trajectory analysis was used to identify patterns in the natural history of asthma and wheezing.

## METHODS

### Study population

From January 1989 to February 1990 in the Isle of Wight (IOW), United Kingdom, the parents of every child (*n* = 1536) were approached to participate in a longitudinal study. After exclusion of adoptions, perinatal deaths, and refusals, 94.8% (1456/1536) of all parents enrolled their newborn. The local research ethics committee approved the study. Informed written parental consent was obtained for all participants at recruitment and again at each follow-up. The IOW birth cohort has been described in detail elsewhere.^6^^,^^7^ Children were followed up at the ages of 1 (94.4%), 2 (84.5%), 4 (83.6%), 10 (94.3%), and 18 years (90.2%). The birth cohort is a dynamic cohort; children who dropped out of 1 exam were free to rejoin the cohort at a later age.

### Questionnaires

Detailed questionnaires, including the International Study of Asthma and Allergies in Childhood (ISAAC) questionnaire,^8^ were completed by parents (or the study subject at age 18 years) at each follow-up. At the 1-, 2-, and 4-year follow-ups, asthma was ascertained by the medical investigator as history of physician-diagnosed asthma plus at least 1 episode of wheezing during the previous 12 months. At the 10- and 18-year follow-ups asthma was defined as having “ever had asthma” and either “wheezing or whistling in the chest during the previous 12 months” or “current treatment for asthma”. Wheezing was defined as wheezing or whistling during the previous 12 months. Because the 1- and 2-year follow-up data were collected during a relatively small time window, we combined them for analytic purposes (reported hereafter as “age 1 or 2 years”).

### Measures of disease occurrence

Period prevalence was determined by dividing the number of cases by the total number of participants in the respective year of follow-up; incidence was defined as the number of new cases divided by the cohort at risk (disease-free in all preceding follow-ups); cumulative incidence was estimated by dividing all cases by those cohort members who participated in the respective and all preceding follow-ups (stable cohort); and remission was defined as number of cases of “outgrown” asthma divided by the cohort who had the disease in preceding follow-ups. Transitions were defined as a change in a person's disease status between 2 consecutive assessments, regardless of whether it was the first such occurrence or whether prior data were missing. There were 3 transition periods: age 1 or 2 to 4 years, age 4 to 10 years, and age 10 to 18 years. Positive transition was defined as the number of cases divided by the study group that was disease-free at the preceding exam. Negative transition was determined accordingly.

### Statistical analysis

All statistical analyses were performed using the SAS statistical package, Version 9.2 (SAS Institute, Cary, NC, USA). For each observation period (age 1 or 2, 4, 10, and 18 years) we used χ^2^ tests with 2-sided *P* values to estimate differences in proportions and rates among boys and girls.

Latent transition analysis (LTA) was performed to identify latent classes of dual occurrence of asthma and wheezing and assess their transition probabilities at different ages (1 or 2, 4, 10, and 18 years), stratified by sex. LTA estimates 3 types of parameters^9^: (1) probabilities of items describing the latent class, conditional on latent status and time, (2) probability of membership in each latent status at each time point, and (3) conditional transition probabilities. The following criteria were used for model selection: the likelihood-ratio *G*^2^ statistic, the Akaike Information Criterion (AIC), and the Bayesian Information Criterion (BIC).

We used semiparametric mixture modeling (PROC TRAJ macro)^10^—which combines the latent growth curve and mixture modeling—to identify developmental trajectories for asthma and wheezing over time (age 1 or 2, 4, 10, and 18 years) separately for boys and girls.^11^ Trajectory parameters were estimated using the maximum likelihood approach built upon a binary logit model.^12^^,^^13^ The best model was taken as the one with the smallest absolute BIC value^14^ and no overlap in the 95% CIs of adjacent trajectories.^12^ The objective of model selection is to summarize the distinctive features as parsimoniously as possible.^11^ Parameter estimates for linear, quadratic, and cubic modeling terms for each subgroup were tested for each trajectory. The PROC TRAJ macro assumes that missing data are missing completely at random.

Because the increase in asthma prevalence at ages 10 and 18 years may be due to a difference in the person who answered the question (parent vs child), we used data from lung function assessments and skin prick testing as objective markers to validate sex differences.

## RESULTS

### Measures of disease occurrence

The period prevalence of asthma among boys and girls ranged from 15.7% to 16.9% and from 11.7% to 19.4%, respectively, during the first 18 years of life (Table 1). At age 10, more boys than girls were affected by asthma (*P* = 0.01). However, at age 18 years there was a switchover in asthma prevalence, ie, more girls than boys were affected (19.4% and 15.9%; respectively; *P* = 0.09). The positive transition of asthma between ages 4 and 10 years was higher among boys (10.5%) than among girls (5.4%; *P* < 0.01). However, the positive transition of asthma from age 10 to age 18 years was higher among girls (10.8%) than among boys (7.1%; *P* = 0.03). No sex differences were detected in asthma remission (Table 1). Between ages 1 or 2 and 4 years, asthma remission was identical to negative transition. On the basis of negative transition, boys were more likely than girls to outgrow asthma between ages 10 and 18 years (*P* = 0.02). Regarding asthma occurrence, measures of prevalence, incidence, cumulative incidence, and remission showed no sex-specific changes between ages 10 and 18 years, whereas positive and negative transitions revealed a sex switchover in asthma prevalence.

**Table 1.** Measures of association to determine asthma occurrence and wheezing episodes from infancy through adolescence (IOW Cohort Study, UK)

	Age 1 or 2 % (*n*/*N*)	Age 4 % (*n*/*N*)	Age 10 % (*n*/*N*)	Age 18 % (*n*/*N*)
**Asthma**				

**Prevalence**				
Both	14.3 (197/1377)	14.9 (181/1214)	14.7 (201/1368)	17.7 (231/1305)
Boys	16.9 (118/700)	15.7 (97/619)	16.9 (118/696)	15.9 (103/646)
Girls	11.7 (79/677)	14.1 (84/595)	12.4 (83/672)	19.4 (128/659)
*P* value	<0.01	0.44	0.01	0.09
**Incidence**				
Both			7.3 (61/838)	8.2 (59/719)
Boys			9.7 (40/413)	6.8 (23/339)
Girls			4.9 (21/425)	9.5 (36/380)
*P* value			<0.01	0.18
**Cumulative incidence**				
Both			16.4 (153/930)	23.5 (203/863)
Boys			19.1 (88/461)	24.4 (102/418)
Girls			13.9 (65/469)	22.7 (101/445)
*P* value			0.03	0.55
**Positive transition**				
Both		9.7 (95/979)	8.0 (78/981)	9.0 (95/1053)
Boys		10.3 (50/486)	10.5 (52/497)	7.1 (36/509)
Girls		9.1 (45/493)	5.4 (26/484)	10.8 (59/544)
*P* value		0.53	<0.01	0.03
**Remission**				
Both			89.5 (77/86)	93.1 (67/72)
Boys			88.7 (47/53)	97.6 (41/42)
Girls			90.9 (30/33)	86.7 (26/30)
*P* value			0.74	0.07
**Negative transition**				
Both		55.2 (90/163)	47.7 (84/176)	32.6 (59/181)
Boys		58.5 (55/94)	47.4 (45/95)	39.4 (41/104)
Girls		50.7 (35/69)	48.2 (39/81)	23.4 (18/77)
*P* value		0.32	0.91	0.02

**Wheezing**				

**Prevalence**				
Both	20.4 (279/1365)	21.4 (261/1218)	18.9 (259/1373)	22.6 (294/1302)
Boys	23.4 (162/691)	23.7 (147/620)	21.5 (150/697)	19.5 (126/646)
Girls	17.4 (117/674)	19.1 (114/598)	16.1 (109/676)	25.6 (168/656)
*P* value	<0.01	0.04	0.01	<0.01
**Incidence**				
Both			11.1 (84/754)	11.6 (71/614)
Boys			12.7 (46/363)	8.4 (24/286)
Girls			9.7 (38/391)	14.3 (47/328)
*P* value			0.19	0.02
**Cumulative incidence**				
Both			23.8 (209/879)	33.1 (268/811)
Boys			27.0 (117/434)	33.2 (130/392)
Girls			20.7 (92/445)	32.9 (138/419)
*P* value			0.02	0.94
**Positive transition**				
Both		13.8 (127/919)	1.9 (108/909)	14.3 (144/1004)
Boys		15.9 (72/454)	13.6 (61/450)	11.3 (55/485)
Girls		11.8 (55/465)	10.2 (47/ 459)	17.2 (89/519)
*P* value		0.07	0.12	<0.01
**Remission**				
Both			83.0 (83/100)	90.1 (73/81)
Boys			84.5 (49/58)	95.7 (45/47)
Girls			81.0 (34/42)	82.4 (28/34)
*P* value			0.64	0.04
**Negative transition**				
Both		48.6 (107/220)	54.5 (139/255)	43.5 (101/232)
Boys		49.6 (61/123)	52.8 (76/144)	50.0 (65/130)
Girls		47.4 (46/97)	56.8 (63/111)	35.3 (36/102)
*P* value		0.74	0.52	0.02

**Asthma combined with wheezing**				

**Prevalence**				
Both	12.9 (176/1365)	14.7 (179/1368)	13.0 (178/1368)	16.7 (216/1296)
Boys	15.3 (106/691)	15.5 (96/619)	15.4 (107/696)	14.4 (92/641)
Girls	10.4 (70/674)	14.0 (83/595)	10.6 (71/672)	18.9 (124/655)
*P* value	<0.01	0.44	<0.01	0.03
**Incidence**				
Both			5.9 (44/742)	4.9 (29/597)
Boys			7.8 (28/359)	3.3 (9/277)
Girls			4.2 (16/385)	6.3 (20/320)
*P* value			0.03	0.09
**Cumulative incidence**				
Both			14.6 (126/863)	21.7 (172/794)
Boys			17.3 (74/427)	22.1 (85/384)
Girls			11.9 (52/436)	21.2 (87/410)
*P* value			0.02	0.75
**Positive transition**				
Both		8.5 (77/904)	6.2 (56/903)	6.6 (64/975)
Boys		9.2 (41/447)	8.7 (39/449)	5.1 (24/47)
Girls		7.9 (36/457)	3.7 (17/454)	7.9 (40/504)
*P* value		0.49	<0.01	0.07
**Remission**				
Both			80.3 (49/61)	93.8 (45/48)
Boys			82.5 (33/40)	96.9 (31/32)
Girls			76.2 (16/21)	87.5 (14/16)
*P* value			0.56	0.21
**Negative transition**				
Both		43.5 (64/147)	40.2 (70/174)	30.8 (49/159)
Boys		48.8 (41/84)	38.3 (36/94)	34.0 (32/94)
Girls		36.5 (23/63)	42.5 (34/80)	26.2 (17/65)
*P* value		0.14	0.57	0.29

*P* values are for sex differences.

At age 4 years, incidence and cumulative incidence were omitted since they estimate the same proportion as that reflected by positive transition.

The period prevalence of wheezing declined over time among boys but increased among girls (Table 1). Up to age 10 years, the prevalence of wheezing was higher among boys than among girls. However, a sex switchover in the prevalence of wheezing occurred by age 18 years. New cases (incidence) of wheezing episodes by age 18 years were more frequent among girls (14.3%) than among boys (8.4%). Analysis of the cumulative incidence of wheezing episodes showed that boys experienced more wheezing until age 10; however, there was no sex difference by age 18 years. Positive transition of wheezing between ages 10 and 18 years was higher among girls (17.2%) than among boys (11.3%), while negative transition from age 10 to age 18 years was higher among boys (50.0%) than among girls (35.3%), as was remission. Only cumulative incidence showed no sex-specific reversal between ages 10 and 18 years. However, positive and negative transitions were informative. Similar results were obtained for asthma combined with wheezing.

### Latent transition probabilities

**Figure 1.**
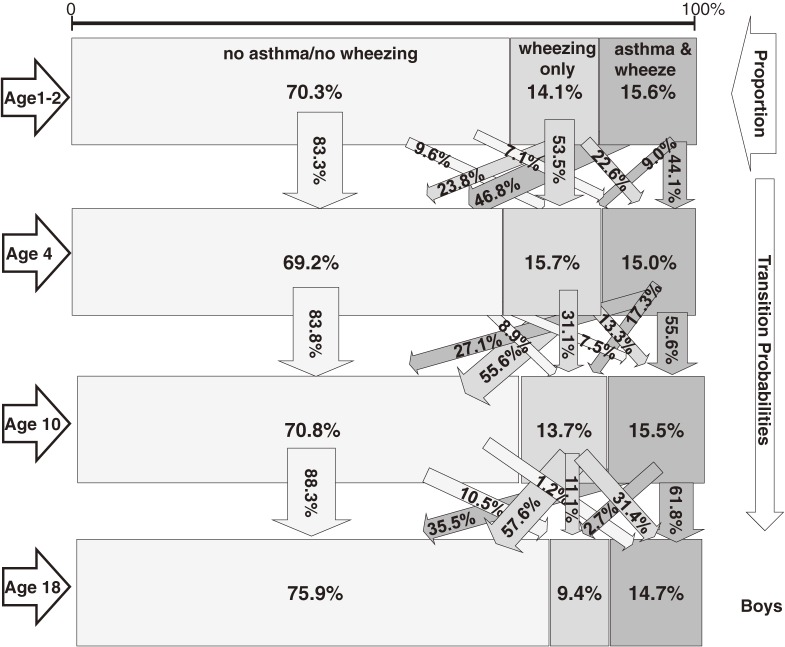
Latent transition for asthma/wheezing episodes among boys in the IOW cohort study, United Kingdom (*n* = 505).

**Figure 2.**
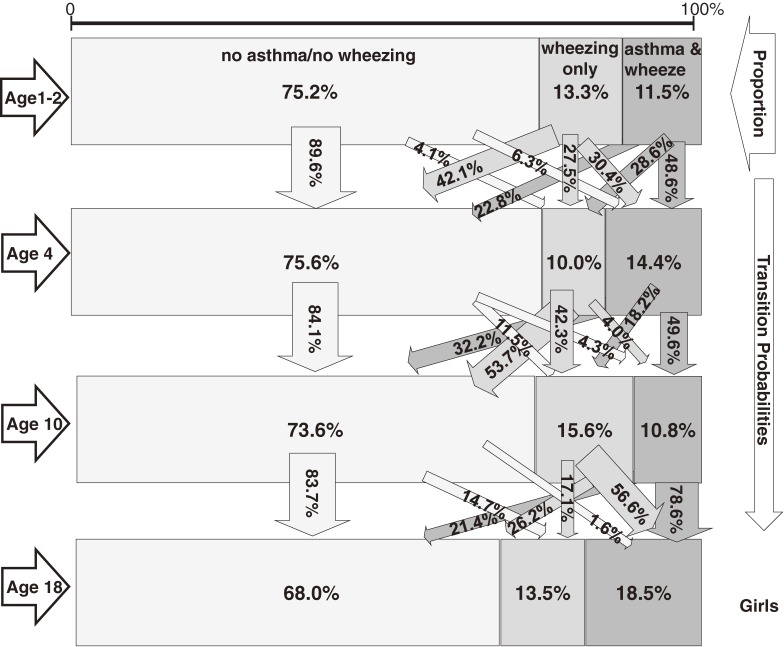
Latent transition for asthma/wheezing episodes among girls in the IOW cohort study, United Kingdom (*n* = 512).

LTA revealed 3 latent statuses at all 4 examinations: “no asthma/no wheezing”, “wheezing only”, and “asthma and wheezing” (Figures 1, 2). Among boys (Figure 1) aged 1 or 2 years, the latent status was no asthma/no wheezing in 70.3%, wheezing only in 14.1%, and asthma and wheezing in 15.6%, and latent status remained unchanged from age 1 or 2 to age 4 years for 83.3% with no asthma/no wheezing, 53.5% with wheezing only, and 44.1% with asthma and wheezing. Boys with asthma and wheezing were most likely to move to disease-free status (no asthma/no wheezing, 46.8%) between ages 1 or 2 and 4 years. Between ages 4 and 10, transitions remained similar for boys with no asthma/no wheezing and those with asthma and wheezing (Figure 1). A large proportion of boys with no asthma/no wheezing did not change status, whereas 56% of those with wheezing only transitioned to the no asthma/no wheezing group. The prevalence of wheezing only declined over time, but the prevalence of asthma and wheezing did not. During the last transition—between ages 10 and 18 years—boys with asthma and wheezing frequently (35.5%) transitioned to disease-free status, but that outflow was replaced by a similar influx from wheezing only status (31.4%).

From age 1 or 2 to age 4 years, a larger proportion of girls than boys with wheezing only transitioned to no asthma/no wheezing (42.1% vs 23.8%) or asthma and wheezing status (30.4% vs 22.6%; Figures 1, 2). Consequently, fewer girls than boys remained in wheezing only status (27.5% vs 53.5%). Sex differences were also obvious for the transition from age 10 to age 18 years. More girls than boys transitioned from wheezing only to asthma and wheezing (56.6% vs 31.4%), and fewer girls than boys moved from that status to no asthma/no wheezing status (26.2% vs 57.6%).

In summary, between ages 1 or 2 and 4 years, girls were more likely than boys to transition from wheezing only to healthy status, whereas between ages 10 and 18 years, girls were less likely than boys to move from wheezing only to healthy status. In contrast to the results for boys, LTA showed that the observed increase in asthma prevalence among girls at age 18 did not result from a transition from no asthma/no wheezing to asthma but rather from a transition from wheezing only to asthma and wheezing status (Figure 2). This was not detectable when status was simply dichotomized as asthma versus no asthma.

### Trajectories

**Figure 3.**
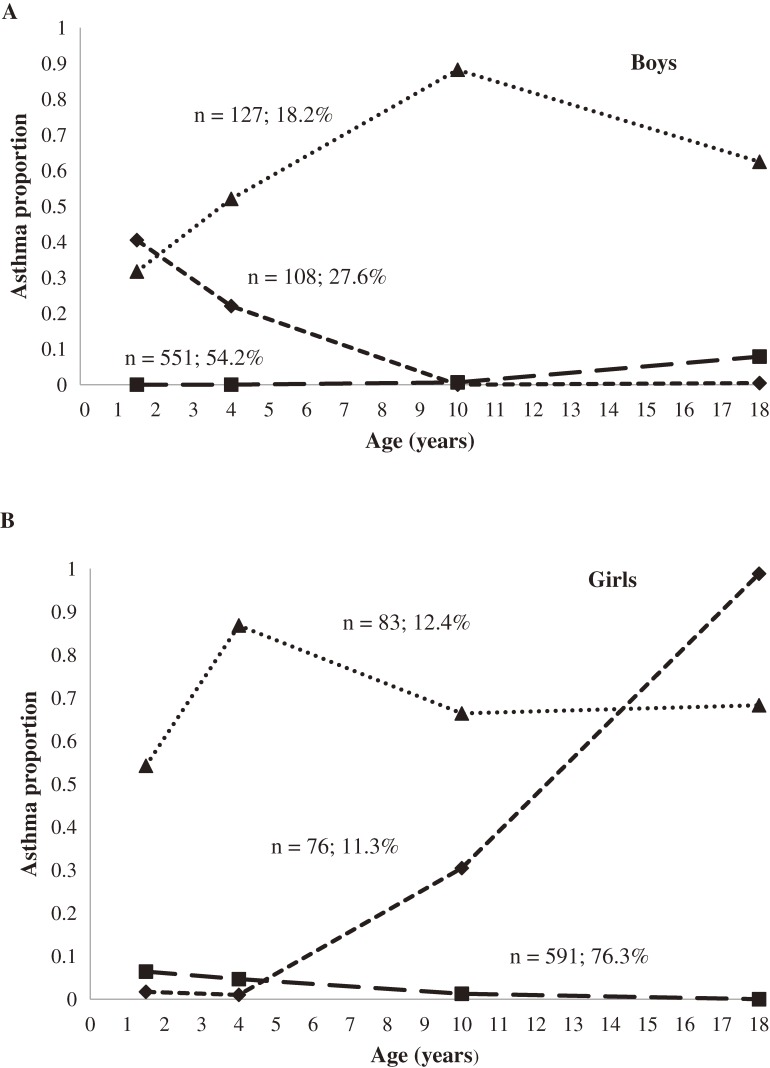
Predicted trajectories of asthma prevalence over time among boys and girls in the IOW cohort study, United Kingdom. (A) Group percentages: triangle (developing asthma): *n* = 127 or 18.2%, diamond (growing out of asthma): *n* = 108 or 27.6%, square (never/infrequent asthma): *n* = 551 or 54.2%. Trajectory analysis of asthma among boys showed that the 3-group model was the best fitting because it had the smallest Bayesian Information Criterion value (−1110.98) and the 95% CIs of adjacent trajectories did not overlap. The 3-group model for asthma, with cubic, linear, and cubic modeling terms, provided a better representation of the data than did other combinations of modeling terms (*P* < 0.05). (B) Group percentages: triangle (growing out of asthma): *n* = 83 or 12.4%, diamond (developing asthma): *n* = 76 or 11.3%, square (never/infrequent asthma): *n* = 591 or 76.3%. As was the case for boys, the 3-group model was selected for girls with asthma, as it had the lowest Bayesian Information Criterion value (−981.10) and the 95% CIs of adjacent trajectories did not overlap. The 3-group model for asthma, with linear, quadratic, and cubic modeling terms, provided a better representation of the data than did other combinations of modeling terms (*P* < 0.05).

To determine whether trajectories of asthma and wheezing had similar patterns over age, we explored various sex-stratified models. In each sex stratum, using the best solutions, we identified 3 trajectories separately for asthma and for wheezing. For boys these were (1) developing asthma (18.2%), (2) growing out of asthma (27.6%), and (3) never/infrequent asthma (54.2%; Figure 3A). For girls, the trajectories were (1) developing asthma (11.3%), (2) intermittent asthma (12.4%), and (3) never/infrequent asthma up to age 18 years (76.3%; Figure 3B). In the comparable trajectory of never/infrequent asthma, there were fewer boys (54.2%) than girls (76.3%; Figure 3). Boys had a growing out of asthma trajectory, while girls had an intermittent asthma trajectory; approximately 70% of participants included in the trajectories had asthma at all 4 time points.

**Figure 4.**
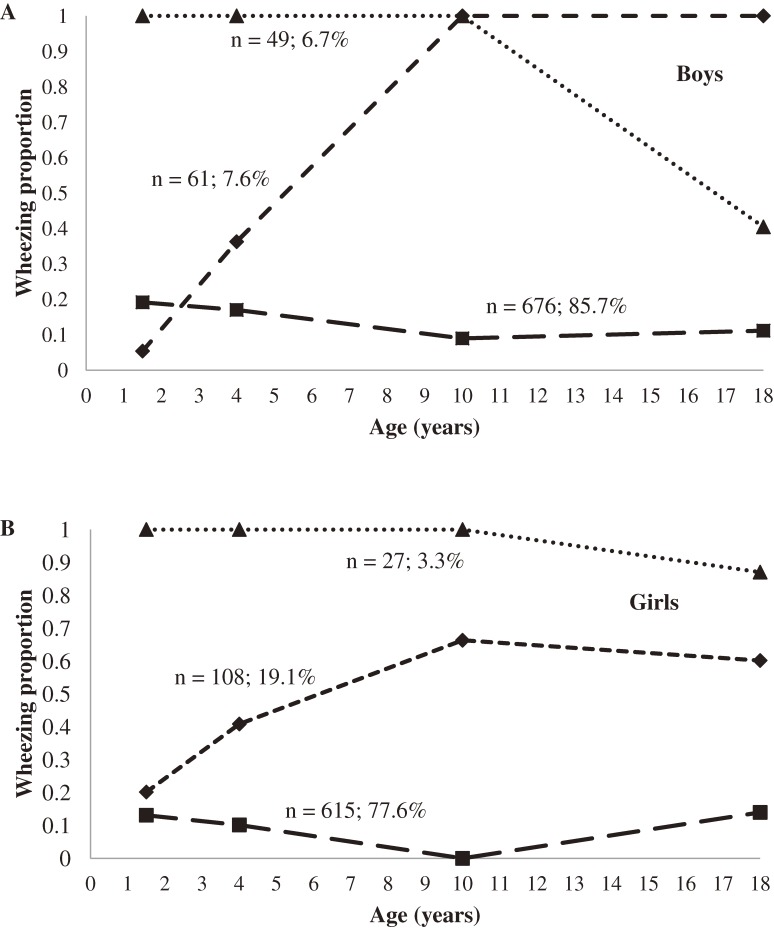
Predicted trajectories of wheezing prevalence over time among boys and girls in the IOW cohort study, United Kingdom. (A) Group percentage: triangle (growing out of wheezing): *n* = 49 or 6.7%, diamond (developing wheezing): *n* = 61 or 7.6%, square (infrequent wheezing): *n* = 676 or 85.7%. Trajectory analysis of wheezing among boys showed that the 3-group model was the best fitting because it had the smallest Bayesian Information Criterion value (−1402.30) and the 95% CIs of adjacent trajectories did not overlap. The 3-group model for wheezing, with linear, cubic, and cubic modeling terms, provided a better representation of the data than did other combinations of modeling terms (*P* < 0.05). (B) Group percentage: triangle (persistent wheezing): *n* = 27 or 3.3%, diamond (developing wheezing): *n* = 108 or 19.1%, square (infrequent wheezing): *n* = 615 or 77.6%. As was the case for boys, the 3-group model was selected for girls with wheezing, as it had the lowest Bayesian Information Criterion value (−1234.86) and the 95% CIs of adjacent trajectories did not overlap. The 3-group model for wheezing, with cubic, cubic, and cubic modeling terms, provided a better representation of the data than did other combinations of modeling terms (*P* < 0.05).

For wheezing, the 3 trajectories in boys were: (1) growing out of wheezing (6.7%), (2) developing wheezing (7.6%), and (3) infrequent wheezing/wheeze-free (85.7%; Figure 4A). The 3 trajectories in girls were (1) persistent wheezing (3.3%), (2) developing wheezing (19.1%), and (3) infrequent wheezing/wheeze-free (77.6%; Figure 4B). More girls than boys seemed to develop wheezing during the course of the study (19.1% vs 7.6%), and a persistent wheezing trajectory was seen among girls (3.3%) but not among boys.

In summary, the relapsing–remitting course of asthma was incorporated into 2 trajectories for boys (the developing asthma and growing out of asthma trajectories; Figure 3A) and 1 trajectory for girls (the intermittent asthma trajectory; Figure 3B). Among boys, the developing asthma trajectory started at age 2, peaked at age 10, and declined thereafter. Among girls, the developing asthma trajectory started at age 10 years and continued up to age 18 years. Among boys, 27.6% “outgrew” asthma, which started at age 1 or 2 years. Regarding wheezing trajectories, the proportion of wheezers remained stable over the course of the study among girls but not among boys. Both sexes showed a developing wheezing trajectory that started at age 1 or 2, peaked at age 10, and persisted thereafter.

Regarding the validity of asthma diagnosis, we compared questionnaire information with the results of lung function assessment and skin prick testing at ages 10 and 18 years. Our concern was that the increase in asthma prevalence among girls at age 18 years could have been due to differences in the person who answered the questions (parent vs child). Such differential reporting would be suspected if the association of self-reported asthma with objective markers, such as allergic sensitization (skin prick tests) and lung function measurements, was diminished among girls who may have over-reported asthma at age 18 years. However, the associations with the results of skin prick tests (Table 2) and lung function assessments (Table 3) were not weaker among girls than among boys. On the contrary, among girls who developed asthma between ages 10 and 18 years, the increases in forced vital capacity (FVC) and forced expiratory volume in 1 second (FEV_1_) were lower than among girls with no asthma (1.54 L vs 1.74 and 1.27 L vs 1.51 L, respectively). Hence, the changes in FVC and FEV_1_ suggest that, as compared with boys, girls who reported asthma at age 18 may have had more-severe asthma. Therefore, the higher prevalence of asthma among girls at age 18 years cannot be explained by over-reporting among girls.

**Table 2.** Adjusted effects of asthma on skin prick test (SPT) positivity^a^ at ages 10 and 18 years in a log-linear regression model

		Skin Prick Test positivity^b,c^			
		
		Age 10 years (*n* = 1034)		Age 18 years (*n* = 852)	
			
		Risk ratio	*P* value	Risk ratio	*P* value
Boys:	asthma	2.80	<0.0001	2.05	<0.0001
	no asthma	reference		reference	
Girls:	asthma	3.03	<0.0001	2.00	<0.0001
	no asthma	reference		reference	

^a^At ages 10 and 18 years SPTs were performed with 12 allergens (house dust mite, cat, dog, *Alternaria alternata*, *Cladosporium herbarium*, grass pollen mix, tree pollen mix, cows’ milk, soya, hens’ egg, peanut, and cod, plus positive and negative controls; ALK, Horsholm, Denmark), following a standardized technique. A positive SPT was defined as a mean wheal diameter at least 3 mm larger than that of the negative control.^20^

^b^As expected, the prevalence of skin prick test positivity significantly increased during adolescence, and the increase in sensitization with age was significantly greater among boys than among girls.^20^ Among children with asthma, a significant difference was found between the proportion of SPT positivity at ages 10 and 18 years (*P* < 0.0001).

^c^Previously we showed that boys and girls in this cohort had similar patterns of sensitization across childhood and adolescence.^20^ More girls were assessed at age 18 years; however, there was no significant association between proportion of follow-up and SPT results, either overall or after stratification by sex.

**Table 3.** Adjusted effects of asthma on lung function^a^ at ages 10 and 18 years in a linear regression model^b^

	Age 10 years		Age 18 years	
		
	Adjusted means	*P* value	Adjusted means	*P* value
	**FEV_1_ (L)**			

Boys	*n* = 488		*n* = 395	
asthma	2.04	0.52^c^	4.42	0.0002^c^
no asthma	2.06		4.66	
Girls	*n* = 490		*n* = 443	
asthma	1.98	0.57^c^	3.30	<0.0001^c^
no asthma	1.99		3.51	

	**FVC (L)**			

Boys	*n* = 488		*n* = 394	
asthma	2.37	0.40	5.39	0.49
no asthma	2.34		5.34	
Girls	*n* = 491		*n* = 443	
asthma	2.27	0.14	3.92	0.35
no asthma	2.23		3.96	

	**FEV_1_/FVC ratio**			

Boys	*n* = 488		*n* = 347	
asthma	0.86	0.01^c^	0.82	<0.0001^d^
no asthma	0.88		0.87	
Girls	*n* = 490		*n* = 412	
asthma	0.87	0.0005^c^	0.84	<0.0001^d^
no asthma	0.89		0.88	

		**FEV_1_ at age 18 minus FEV_1_ at age 10 (*n* = 589)**		

Boys (*n* = 269)				
positive transition of asthma^e^		2.54		0.78
no asthma		2.57		
Girls (*n* = 320)				
positive transition of asthma^e^		1.27		0.0004
no asthma		1.51		

		**FVC at age 18 minus FVC at age 10 (*n* = 588)**		

Boys (*n* = 268)				
positive transition of asthma^e^		3.05		0.46
no asthma		2.97		
Girls (*n* = 320)				
positive transition of asthma^e^		1.54		0.01
no asthma		1.74		

		**FEV_1_/FVC ratio at age 18 minus FEV_1_/FVC ratio at age 10 (*n* = 589)**		

Boys (*n* = 269)				
positive transition of asthma^e^		0.006		0.17
no asthma		−0.010		
Girls (*n* = 320)				
positive transition of asthma^e^		−0.025		0.24
no asthma		−0.012		

^a^Lung function measurements were performed at ages 10 and 18 years using a Koko Spirometer and software with a portable desktop device (both from PDS Instrumentation, Louisville, KY, USA), according to American Thoracic Society guidelines.^24^

^b^Adjusted by child sex and height.

^c^Adjusted by child height.

^d^Adjusted by child height and age of puberty.

^e^Children with a positive transition in asthma status from age 10 to age 18 years as compared with children without asthma at ages 10 and 18 years.

## DISCUSSION

Prevalence, incidence, cumulative incidence, and remission are not always sensitive measures of time-specific changes in asthma occurrence, but positive and negative transitions proved to be sensitive measures in the present study. For wheezing, measures of prevalence, incidence, and remission detected a statistically significant sex switchover between ages 10 and 18 years; however, positive and negative transition probabilities were more informative. The advantage of a transitions measure is that it can be applied to dynamic cohorts and is independent of restrictions imposed by stable cohorts. If disease prevalence changes, there is a need to understand what dynamics contributed to the change. To this end, positive and negative transition probabilities better explain the nature of such changes. Latent transition analyses provide novel information on both stability and change in a dynamic cohort, ie, one from which subjects may drop out but later rejoin. Trajectory analysis identified dynamic patterns with a relapsing–remitting course. In comparison to LTA, trajectory analysis does not directly identify a switchover in prevalence among boys and girls. To detect a switchover, dynamic trajectories and changing proportions of symptoms need to be identified.

We have shown that asthma/wheezing is not a stable phenotype and that movement from asthma/wheezing to no asthma/wheezing is frequent. During the transitional period of adolescence, a sex switchover from male predominance of asthma in childhood to female predominance by age 18 was observed, which agrees with findings in other populations.^15^^–^^17^ Surprisingly, the increase in asthma among girls at age 18 years was not the result of girls without wheezing developing new asthma but of girls with wheezing transitioning to asthma.

Regarding information bias, nearly identical definitions of asthma and wheezing were used from birth to age 18 years. The definition of asthma at ages 10 and 18 years included the criterion of asthma treatment, which was not strictly applied to define asthma at younger ages (ie, at 1 or 2 and 4 years). We used slightly different diagnostic criteria because asthma diagnoses for young children may include infectious responses and because asthma treatment varies in younger children and is thus not recommended for all children with an asthma diagnosis.^18^^,^^19^ Asthma prevalence was similar at the first 3 time points (approximately 14.5%). An increase in prevalence occurred between ages 10 and 18 years but not between ages 4 and 10 years, that is to say, when treatment was added to the definition of asthma (Table 1). Hence, a minor change in the definition of asthma over time did not influence the interpretation of our results.

Ascertainment of asthma status at a specific age was based on 2 components: a patient reporting that they had a history of asthma (“ever had asthma”) and concurrent information. A history of asthma reflects either a past condition or a new episode. As concurrent criteria, wheezing and/or asthma treatment were required. A comparison of observational data on allergic sensitization^20^—a marker for allergic asthma—with lung function results^21^^,^^22^ showed that over-reporting was unlikely among girls.

When assessing the natural history of diseases in longitudinal studies, a major source of bias is loss to follow-up. For instance, in the British 1958 Birth Cohort, follow-up to age 23 years was less than 45%.^23^ If prior disease status increased retention and a stable cohort is analyzed, then cumulative incidence is overestimated. Conversely, if no information is provided on prior disease status and retention, then information on natural history may be biased, since the dynamic of late disease onset becomes underestimated. Because follow-up in our cohort was excellent over the 18-year period of the study, we were able to compare the characteristics of different disease measures. For example, in our data, cumulative incidence did not identify the sex switchover in asthma prevalence between ages 10 and 18 years (Table 1).

Disease prevalence at different time points may provide initial information on potential disease patterns. Cumulative incidence seems to be the least appropriate measure to identify changes in a specific time window, since events are cumulated over time. Therefore, if prior events predominate numerically, the aggregation of events makes cumulative incidence insensitive for life-period–specific changes.

The problem of not considering the difference between incidence and positive transition, or that between remission and negative transition, was evident in a recent work on gender shift in asthma.^5^ Incident asthma was defined as presence of asthma at the time of a specific survey in a person without asthma at the previous survey, which is the definition of transition. Using this definition for incidence leads to confusion with regard to true incidence (ie, a first disease episode). Since asthma waxes and wanes, it is unlikely that participants who reported not having asthma in the aforementioned study were truly disease-free. In the Isle of Wight birth cohort, for example, among respondents at age 18 years, only 39% of those with asthma at age 1 or 2 years (*n* = 86), and 60% of those who had asthma at age 4 years (*n* = 70), reported that they had asthma before (data not shown). Therefore, use of the terms positive and negative transition is necessary to avoid confusion and provide comparable measures of disease frequency among different studies.

In trajectory analysis, most of the identified trajectories for asthma and wheezing were stable over time: either symptoms or no symptoms. Patterns that incorporated the dynamic of asthma showed an increasing or a decreasing proportion of members with asthma. A disadvantage of trajectory analysis is that it cannot identify switchovers between the distinct, but related, phenotypes of asthma and wheezing.

LTA is suitable for describing the probabilities of transitions in disease status for a dynamic population over time. LTA focuses on transition probabilities between 2 time points; it does not show how individuals track over multiple transitions. Nevertheless, this method yielded a parsimonious yet refined summary of the heterogeneity of asthma and wheezing. By using LTA, we were able to reveal sex-specific differences in transition in our cohort.

We compared the results for different measures of disease occurrence and their ability to detect time-specific changes in diseases that wax and wane. In this approach, we did not consider the impact of risk factors. However, evaluation of risks will vary with the measures of disease occurrence. Applying positive and negative transitions to epidemiologic research is important not only for asthma and wheezing but also for a variety of diseases with a remitting–relapsing course such as musculoskeletal disorders and pregnancy-related illnesses. In addition, the use of positive and negative transitions is important for transition periods throughout life—not just puberty (as was examined in this work)—to address disease risks related to work life, menopause, and retirement.

In conclusion, classical measures of disease (incidence, cumulative incidence, and remission) are not sufficiently informative and cannot be properly applied to yield conclusions about time-specific changes in disease status. In elucidating changes in prevalence, positive and negative transition probabilities are helpful in identifying disease dynamics. In addition, transition probabilities do not require a stable cohort: they can be estimated in a dynamic cohort, thereby providing a meaningful understanding of disease dynamics. Hence, transition analyses impose few restrictions on data. In particular, latent transition analyses can use multiple statuses (eg, asthma and wheezing) and can thus generate novel information regarding the transition between disease statuses.
